# Proportion and associated factors of the utilisation of complementary and alternative medicine exclusively in a hospital in Bangladesh

**DOI:** 10.1186/s12906-022-03709-8

**Published:** 2022-08-26

**Authors:** Md. Shahjalal, Jeff Gow, Md. Ashfikur Rahman, Md. Jakir Hossain, Md. Nafiul Alam Khan, Md. Sazzadul Alam, Ahmed Hossain, Rashidul Alam Mahumud

**Affiliations:** 1 Department of Public Health, North South University, Dhaka, Bangladesh; 2 Research Rats, Dhaka, Bangladesh; 3 Government Unani &, Ayurvedic Medical College & Hospital, Dhaka, Bangladesh; 4 School of Business and Centre for Health Research, University of Southern Queensland, Toowoomba, QLD Australia; 5 School of Accounting, Economics and Finance, University of KwaZulu-Natal, Durban, South Africa; 6 Development Studies Discipline, Khulna University, Khulna, Bangladesh; 7 Department of Statistics, Khulna University, Khulna, Bangladesh; 8 Department of Health Services Administration, College of Health Sciences, University of Sharjah, Sharjah, United Arab Emirates; 9 NHMRC Clinical Trials Centre, Faculty of Medicine and Health, The University of Sydney, Camperdown, New South Wales Australia

**Keywords:** Complementary and alternative medicine, Conventional medicine, Bangladesh

## Abstract

**Background:**

Complementary and alternative medicine (CAM) has played a critical role in ensuring universal access to basic health care services around the world. In Bangladesh, conventional medicine is a common approach for health care practices, yet, due to Bangladesh’s high out-of-pocket payment, millions of people utilise CAM-based healthcare services for illnesses. In Bangladesh, there is a scarcity of data on how CAM is perceived and utilised. The goal of this study was to determine the proportion and correlates of the utilisation of CAM among patients visiting a tertiary level hospital, in Bangladesh.

**Methods:**

A cross-sectional survey involving 1,183 patients who received health care from a hospital in Bangladesh was interviewed for this study. The associated factors on utilising CAM were identified using multivariable logistic regression analysis.

**Results:**

Thirty-three percent of patients utilised CAM exclusively to treat their illnesses, whereas the rest utilised conventional medicine before CAM. Young adult patients aged 26 to 45 years (AOR = 6.26, 95% CI:3.24–12.07), patients without education (AOR = 2.99, 1.81–4.93), and married patients (AOR = 1.79, 1.08–2.97) were the most likely to be only CAM users. The most common reasons for using CAM were belief in its effectiveness, less adverse effects, affordability and lower costs.

**Conclusion:**

In Bangladesh, CAM plays a significant role in health care delivery, with high-levels of patient satisfaction and health benefits. Patients who are older and have a higher level of education are more hesitant to use CAM for their illness, yet CAM has the potential to play a significant role in reducing hospitalisation by providing high reliability and low costs.

**Supplementary Information:**

The online version contains supplementary material available at 10.1186/s12906-022-03709-8.

## Introduction

Complementary and alternative medicine (CAM) refers to a wide range of products and treatments that are not currently regarded to be part of mainstream medicine. Naturopathy, herbal, homeopathic, ayurvedic, unani, and traditional Chinese medicines are examples of CAM [1, 2]. The CAM has been a popular way to meet people's basic healthcare needs for centuries [1]. This still holds for one-third of the world's population who lack access to conventional medicine [3]. In these circumstances, millions of individuals rely on CAM and its practitioners for their primary healthcare. Often it is their only option due to a lack of access to conventional healthcare, geographic isolation, and high conventional healthcare costs [2, 3]. Although conventional medical access has improved rapidly in recent decades the use of CAM is also increasing globally in illness prevention, control, and management [1–3].

A substantial percentage of people in developed countries use CAM [4–7], and this trend is also seen in developing countries like Bangladesh [8–10]. For example, 29.5% of people in the USA [4], 41.1% in the UK [5], 75% in Australia [6] and 76% of Japanese people [7] use CAM for their primary health care services. Similar to this, CAM is widely used in developing countries such as India (70%) [8], Pakistan (70–80%) [9], and Sri Lanka (76%) [10]. A recent study reported that people in Southeast Asia used CAM treatments primarily, from 20 to 97% [11]. While conventional medicine has made significant progress in Bangladesh, a considerable number of people uses various forms of CAM treatment for their primary health care [12–14]. According to a recent survey, 32.8% of patients with chronic disease used some form of CAM for their chronic disease management in Bangladesh [15]. Another recent study from Bangladesh reported that 33% of diabetic patients in the northern part of the country used some different types of CAM to manage or treat their condition [16].

A number of studies have documented some potential predictors of CAM usage such as age [1, 4, 15–17], gender [1, 4, 13, 15, 18], religion [1, 8], education [4, 13, 15, 16, 18, 19], marital status [1, 4, 8, 13, 15], residency [12, 13, 15, 16], occupation [6, 13, 15], and household income [1, 4, 16, 19] in both developed and developing countries. The reason why patients choose to use CAM have been much discussed, but not fully understood. The self-reported reasons to use CAM vary in the literature, including less side effects [7, 8, 15], better efficacy [5, 7, 13, 15, 20], dissatisfaction with conventional medicines [7, 18, 19], and the availability and lower costs of CAM [7, 13, 15, 20].

The health care system of Bangladesh has many challenges and barriers, including low service coverage due to limited resources and institutional limitations, high out-of-pocket (OOP) contributions to healthcare, and lack of education among the general population [20, 21]. Equity in health and quality of care are essential pillars for promoting universal health coverage (UHC), which are highly questionable in the Bangladeshi health sector [22–25]. Providing accessibility, quality care, and sustainable financing in healthcare services need to be prioritised in Bangladesh to achieve Sustainable Development Goal (SDG) 3 which promotes healthy lives and well-being [26].

To achieve UHC, CAM can play a vital role in improving health equity and quality of care at the health facility level in Bangladesh [12, 14, 25]. Bangladesh has a wide variety of indigenous plants and indigenous herbal medicine systems are an essential part of the primary healthcare system [13, 14]. Ayurvedic, unani, homeopathy, herbal and naturopathy have been commonly practiced as CAM treatments in Bangladesh [12–15]. As the CAM medical facilities co-exist with conventional medicine systems in Bangladesh, people may use medicine from one system exclusively or acquire treatment from each healthcare system and use it simultaneously or sequentially [14, 15]. Previous studies noted this kind of medical pluralism among patients in this country [13–15]. Although CAM is not considered a part of mainstream healthcare systems, it is used in Bangladesh as an alternative to conventional medicine [13–16]. The utilisation of CAM by patients for treating their illnesses or diseases has been extensively explored elsewhere, but this type of research is lacking in Bangladesh. Two studies in Bangladesh investigated CAM utilisation among patients with chronic diseases [15, 16]. While the studies carried out in Bangladesh move our understanding of CAM use with chronic illnesses forward, these studies did not focus on the utilisation of CAM for acute diseases, which can influence the prevalence and associated factors. By exploring the proportion and associated determinants of CAM utilisation, we can find ways to incorporate CAM into the healthcare system and plan appropriate evidence-based strategies to combat diseases and suggest to healthcare policymakers in improving CAM treatment. Little attention has been paid to investigate the distribution of CAM healthcare utilisation exclusively and associated determinants of CAM utilisation among patients with various health problems in a tertiary care CAM hospital in Dhaka, Bangladesh.

As a result, the current study seeks to determine the proportion of exclusive CAM users among patients in a tertiary care CAM hospital in Dhaka, Bangladesh and their associated variables.

## Methods

### Study design and setting

This study design was a hospital-based cross-sectional survey in Bangladesh. Between December 2019 and May 2020, the survey was conducted among patients who utilised outpatient healthcare services at the Government Unani and Ayurvedic Medical College Hospital in Dhaka, Bangladesh. This hospital is the only tertiary care CAM hospital in Bangladesh.

### Sample size determination and procedure

The required sample size was calculated using the following formula using a 61.5% prevalence of CAM utilisation [17], with a 95% level of confidence and a 5% margin of error.

$$n=\frac{{Z}^{2}\times P\times Q}{{d}^{2}}$$

where n is the appropriate sample size, *P* = 0.615, Q = 1-*P* = (1–0.615), Z = 1.96, and d = 0.05. As a result, a minimum sample size of about 364 is required. Finally, for our study, we gathered data from 1,183 patients.

### Data collection tools and techniques

The survey questionnaire was developed based on: (i) the research objectives and research questions (ii); existing literature and (iii) the authors’ expertise. Before finalising the survey questionnaires: pre-testing and piloting, expert opinion was incorporated in the questionnaire. Patients who sought treatment at the study hospital during data collection were eligible to participate. We conveniently surveyed patients immediately after completing their CAM healthcare visit. Before conducting the survey, the study objectives and benefits were explained to the patients. Patients were informed about the intent of the research and asked to participate by responding to the structured questionnaire (Supplement 1).

Data were obtained either via a face-to-face interview with undereducated or through the self-administration of a questionnaire for those who were literate. Two skilled medical students at the hospital administered the questionnaires to patients. We excluded patients who refused to give written consent and patients with psychiatric disorders by asking if they took medication for their symptoms. Finally, data from 1,183 patients (response rate was 96%) were gathered for analysis, and the flowchart of data collection is given in Supplement 2.

### Data analysis

Categorical variables were described using frequencies and percentages and continuous variables were presented in averages and standard deviations. The utilisation of CAM was determined by comparing socio-demographic data from CAM users to non-users using Pearson’s chi-square test. Multivariable logistic regression model was performed to identify the socio-demographic factors that have a relationship with the use of CAM exclusively. Based on only separated explanatory variables, an unadjusted regression analysis was performed. The *p*-values less than 0.05 in the analysis were considered significant at 95 percent confidence interval. Data from the completed questionnaires were coded and analysed using the Statistical Package for Social Sciences (SPSS) for Windows, Version 26.

## Results

### Participant’s characteristics

This study included a total of 1,183 patients (Table 1) The majority of the participants were between the ages of 18 and 25, female (64.9%), married (70.5%), Muslim (93.7%), and 33.3% had completed university education. A significant proportion of patients (34.7%) were from the highest income quintile. The majority of patients (89.4%) were from nuclear families and resided in urban areas (71.3%). Among patients aged 65 and up, 76.5% had never used CAM before visiting the hospital and 60.2% female patients ever used CAM. Approximately one-thirds of Muslim patients sought CAM treatment, but 80.7% of people with a university education had never used CAM, and 80.5% of high-income people never used CAM before visiting to the hospital.

**Table 1:** Distribution of patient’s characteristics (*n* = 1,183)

Characteristics	Number of patients, n (%)	Utilisation of CAM, n (%)		*p*-value
		**no**	**yes**	
**Age (year)**				
< 18	249(21.0)	195(78.3)	54(21.7)	< 0.001
18–25	283(23.9)	182(64.3)	101(35.7)	
26–45	218(18.4)	86(39.4)	132(60.6)	
46–65	169(14.3)	112(66.3)	57(33.7)	
65 +	264(22.3)	202(76.5)	62(23.5)	
**Gender**				
Male	415(35.1)	315(75.9)	100(24.1)	< 0.001
Female	768(64.9)	462(60.2)	306(39.8)	
**Religion**				
Muslim	1108(93.7)	733(66.2)	375(33.8)	0.186
Others	75(6.3)	44(58.7)	31(41.3)	
**Level of education**				
No education	314(26.5)	168(53.5)	146(46.5)	< 0.001
Primary	270(22.8)	170(63.0)	100(37.0)	
Secondary	205(17.3)	121(59.0)	84(41.0)	
Tertiary	394(33.3)	318(80.7)	76(19.3)	
**Marital status**				
Single	349(29.5)	267(76.5)	82(23.5)	< 0.001
Married	834(70.5)	510(61.2)	324(38.8)	
**Residence**				
Urban	1058(89.4)	703(66.4)	355(33.6)	0.107
Rural	125(10.6)	74(59.2)	51(40.8)	
**Family type**				
Nuclear	844(71.3)	584(69.2)	260(30.8)	< 0.001
Joint	339(28.7)	193(56.9)	146(43.1)	
**Employment status**				
Unemployed	165(13.9)	101(61.2)	64(38.8)	< 0.001
Informal workers	522(44.1)	295(56.5)	227(43.5)	
Formal employee	173(14.6)	137(79.2)	36(20.8)	
Business	81(6.8)	70(86.4)	11(13.6)	
Students	209(17.7)	155(74.2)	54(25.8)	
Others	33(2.8)	19(57.6)	14(42.4)	
**Income quintiles**				
Q_1_ (< 10,000)	158(13.4)	126(79.7)	32(20.3)	< 0.001
Q_2_ (10,000–19,000)	251(21.2)	136(54.2)	115(45.8)	
Q_3_ (20,000–29,0000)	204(17.2)	100(49.0)	104(51.0)	
Q_4_ (30,000–39,0000)	160(13.5)	85(53.1)	75(46.9)	
Q_5_ (≥ 40,000)	410(34.7)	330(80.5)	80(19.5)	

Note: Q1 lowest 20% and Q2 highest 20%; *CAM* Complementary and Alternative Medicine*, p-*value = probability value, *p*-value was derived from the chi-square test

**Fig. 1:**
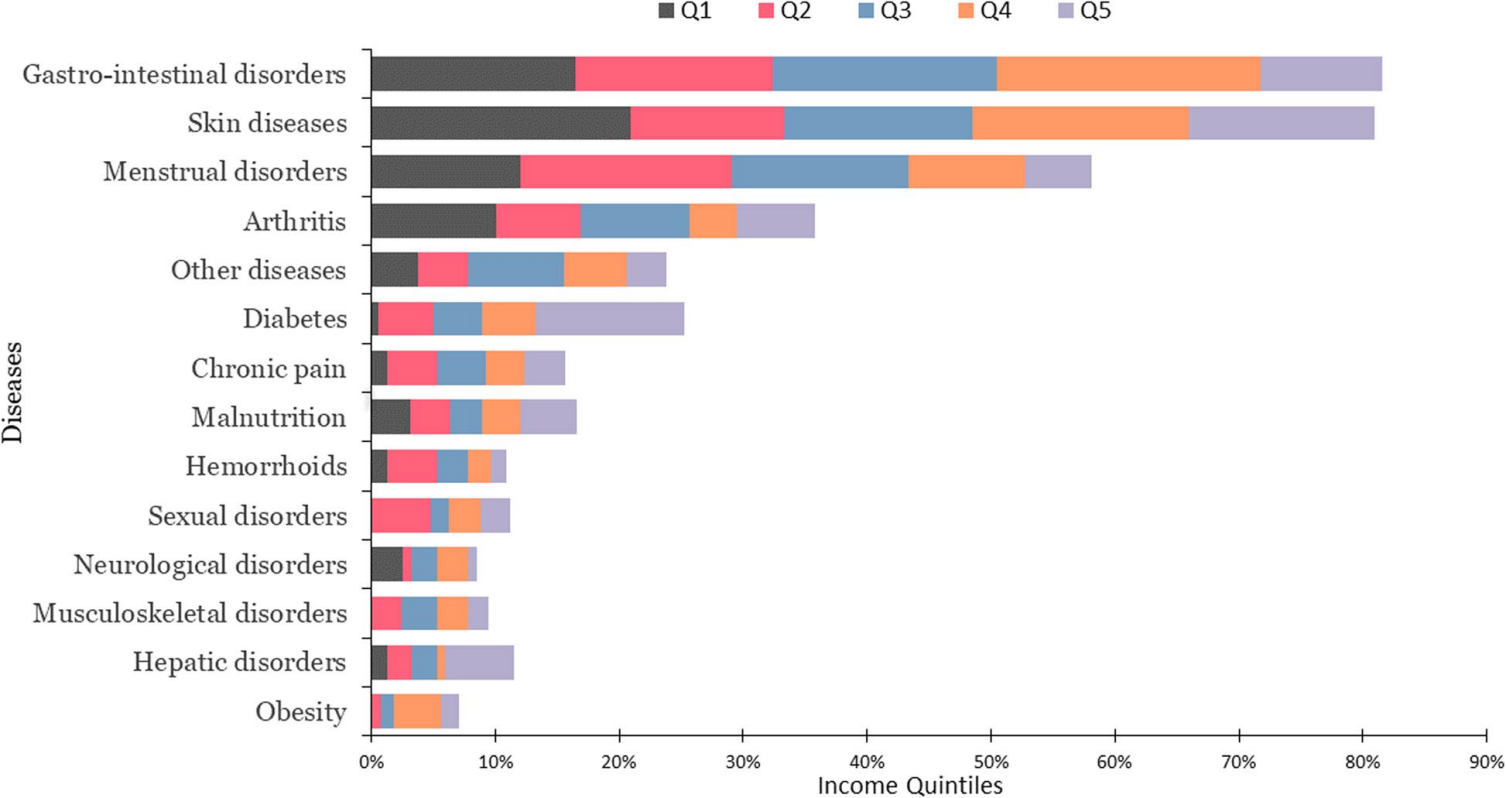
Patients with diseases according to income quintile

There were seventeen different types of health issues that made the patients visited to the hospital. The four most common health ailments for which CAM was utilised were gastrointestinal problems, skin problems, respiratory infections, and menstrual abnormalities (Fig. 1). About 33% of patients utilised CAM exclusively, while 67% used conventional medicine for their illnesses before using CAM.

### Associated factors of using CAM exclusively

Table 2 shows the adjusted model outputs. Patients aged 26–45 years had a substantially higher use of CAM (AOR = 6.26, CI: 3.24 -12.07; *p* < 0.001), followed by the age group 18–25 years (AOR = 2.62, 95% CI: 1.51—4.54; *p* = 0.001), and for the 46–65 years age group (AOR = 2.39, 95% CI: 1.18—4.84; *p* = 0.016). Patients with no or little education were 2.99 and 2.30 times more likely to use CAM than those with the highest level of education, respectively. Married participants were more likely to use CAM (AOR = 1.79, CI:1.08—2.97, *p* = 0.022) than those who were unmarried. The probabilities were 2.18 and 2.34 times higher in patients in the third- and fourth-income quartiles to use CAM than persons in the highest income quantile. Alternatively, patients who were involved in business were 78% (AOR = 0.22; 95% CI: 0.120–0.51; *p* =  < 0.001) or were formally employed 48% (AOR = 0.52, CI:0.29–0.95, *p* = 0.034) were less likely to use CAM. Surprisingly, people from the lowest income quantile were 51% (AOR: 0.49, 95% CI: 0.28–0.88, *p* = 0.016) less likely to utilise CAM than the highest-income patients.

**Table 2:** Association between CAM utilisation and patient characteristics

Characteristics	Unadjusted model		Adjusted model	
	**OR (95% CI)**	***p*-value**	**OR (95% CI)**	***p*-value**
**Age (year)**				
< 18 (= ref)				
18–25	2.00(1.36–2.95	< 0.001	2.62(1.51–4.54)	0.001
26–45	5.54(3.69–8.32)	< 0.001	6.26(3.24–12.07)	< 0.001
46–65	1.84(1.19–2.85)	0.007	2.39(1.18–4.84)	0.016
65 +	1.10(0.73–1.68)	0.627	1.61(0.80–3.24)	0.176
**Gender**				
Male (= ref)				
Female	2.09(1.59–2.72	< 0.001	1.17(0.83–1.63)	0.361
**Religion**				
Muslim	0.73(0.45–1.17)	0.188	0.71(0.41–1.21)	0.213
Others (= ref)				
**Level of education**				
No education	3.64(2.60–5.08)	< 0.001	2.99(1.81–4.93)	< 0.001
Primary	2.46(1.73–3.49)	< 0.001	2.30(1.40–3.78)	0.001
Secondary	2.91(1.99–4.220	< 0.001	2.26(1.41–3.63)	0.001
Tertiary (= ref)				
**Marital status**				
Single (= ref)				
Married	2.07(1.55–2.74)	< 0.001	1.79(1.08–2.97)	0.022
**Residence**				
Urban (= ref)				
Rural	1.36(0.93–1.99)	0.108	0.78(0.48–1.26)	0.318
**Family type**				
Nuclear (= ref)				
Joint	1.69(1.31–2.20)	< 0.001	1.33(0.97–1.83)	0.072
**Employment status**				
Unemployed (= ref)				
Informal workers	1.21(0.84–1.73)	0.287	0.67(0.42–1.06)	0.094
Formal employee	0.41(0.25–0.67)	< 0.001	0.52(0.29–0.95)	0.034
Business	0.24(0.12–0.50)	< 0.001	0.22(0.120–0.51)	< 0.001
Students	0.55(0.35–0.85)	0.008	1.32(0.73–2.38)	0.353
Others	1.16(0.54–2.48)	0.697	0.58(0.24–1.42)	0.238
**Income quintiles**				
Q1 (Lowest 20%)	1.04(0.66–1.65)	0.842	0.49(0.28–0.88)	0.016
Q2	3.48(2.46–4.94)	< 0.001	1.53(0.96–2.44)	0.072
Q3	4.29(2.97–6.19)	< 0.001	2.18(1.38–3.44)	0.001
Q4	3.64(2.4–5.4)	< 0.001	2.34(1.45–3.76)	< 0.001
Q5 (Highest 20%) (= ref)				

Note: *OR* Odds ratio, *CI* Confidence interval

The study hospital offers seven different types of CAM treatment**.** Ayurveda (48%), Unani (45%) and other therapies (7%) like acupressure, acupuncture, yoga, and cupping were the most often used CAM therapies in the hospital (Supplement 3).

**Fig. 2:**
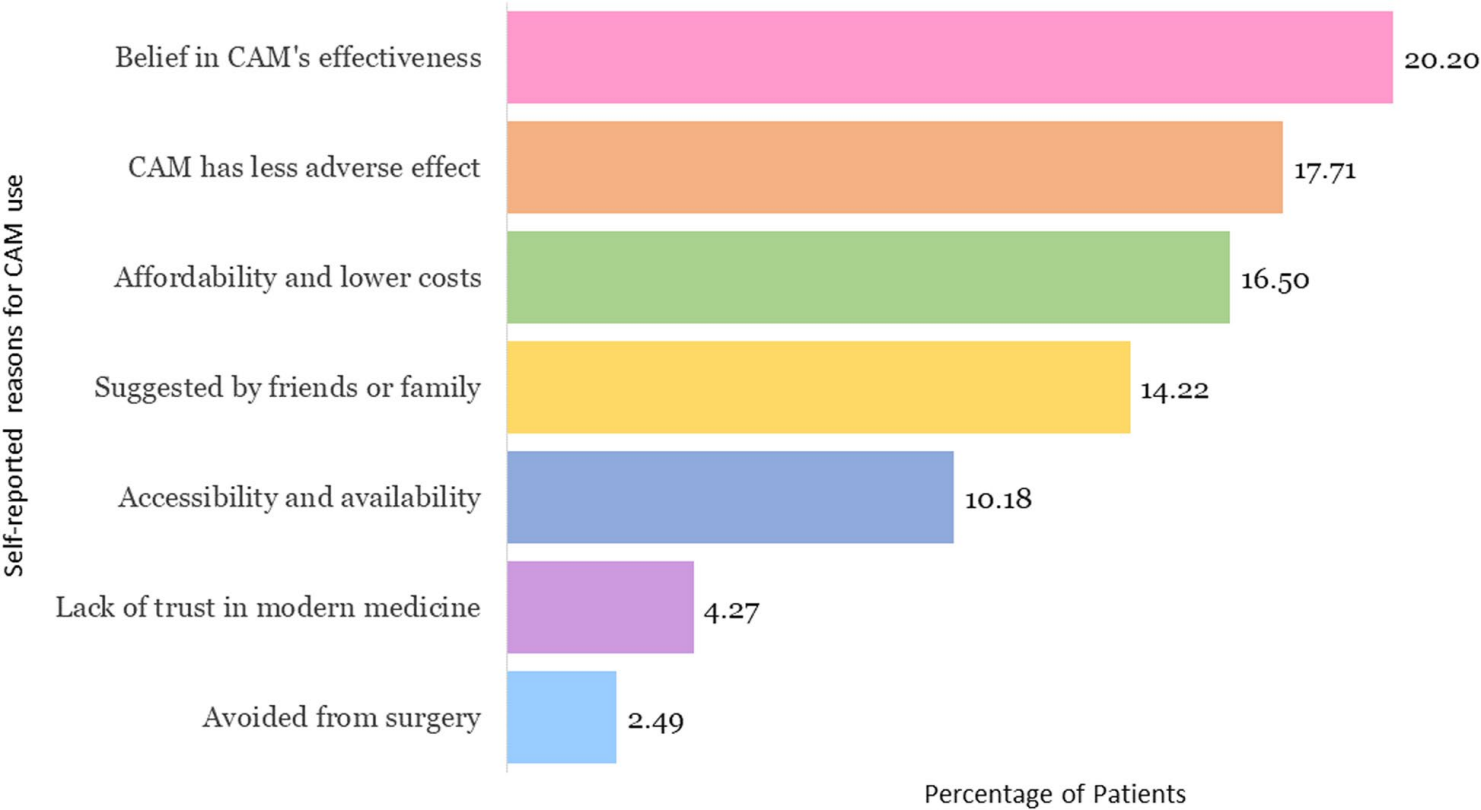
Reasons for using CAM * multiple responses

The reasons why patients chose CAM were investigated and the results are shown in Fig. 2.

The patients reported several reasons for utilising CAM. The most common explanations were: 20.2% believed in its effectiveness,17.7% believed CAM had less adverse effects, and 16.5% used CAM due to its affordability and lower costs.

For patients who took CAM before, we asked them about their satisfaction with CAM treatment. Most of the patients (78%) reported that they were satisfied with CAM treatment for their illnesses. When asked about their willingness to recommend CAM for related health problems, more than half (65%) of the patients reported that they would advise others to use CAM (Supplement 4).

## Discussion

This study focused on the sociodemographic aspects of CAM utilisation in a tertiary care CAM hospital in Bangladesh, as well as the relevant considerations for CAM utilisation. Age, education, marital status, occupation, and income level were positively associated to the usage of CAM. The preference for CAM was highest among middle-aged patients, which is similar to the findings of previously published studies elsewhere [4, 15, 22]. One probable reason is that as a result of their health-seeking behaviours, people have a proclivity to seek out any treatment that would help them improve their health to seek out any treatment that would help them improve their health [13].

Patients with no or school education are more likely to use CAM than those with higher levels of education. However, prior studies have shown that those with higher education and more economically affluent are more inclined to use CAM [4, 15, 17, 19]. This could be because well-educated and financially secure patients are more motivated to look into alternative remedies and ways to cope with their sickness and medication side effects. However, in Bangladesh, poor and non-educated patients often have insufficient money to purchase advanced medical treatment from well-equipped conventional hospitals or clinics [21, 27, 28]. According to the findings, married patients were more likely to used CAM alone than unmarried patients, although a previous study in Bangladesh found that single patients were more likely to utilise CAM alone than married patients [15]. This disparity in results can be attributed to contextualisation and cultural backgrounds. Another factor could be that married women often rely on their husbands for health-seeking behaviour in a male-dominated and patriarchal society like Bangladesh [27].

Patients who own their business or work in a monthly paid-job are less likely to use CAM which contrary to earlier findings [6]. In contrast to a study in India [8] and Pakistan [9], this study found that patients with relatively high-incomes were more likely to utilise CAM. This is similar with prior studies conducted in the USA [4], Ethiopia [19], Nepal [17], and Bangladesh [15]. This could be due to the fact that people with high salaries are more likely to seek out alternatives for their health care and well-being. Although a recent study reported that regardless of socioeconomic status, both the lowest and the highest socioeconomic groups in China exhibited a substantial preference for CAM therapies [29].

This study found that the most common reasons for utilising CAM were: (i) believed in its effectiveness, (ii). perceived less adverse effects, (iii). affordability and lower costs, and (iv). suggested by family and friends. These findings are confirmed by previous studies [4, 5, 8, 9, 15, 30]. The development of CAM treatment is predictable on a broad base of quality research. There is momentum now to expand beyond basic clinical and experimental research to a joint public health program alongside conventional medicine. Interestingly, most patients (78.3%) were satisfied with CAM, and they want to recommend it to others (65.7%) which is in line with attitudes expressed elsewhere in the UK [5], Ethiopia [19] and Bangladesh [12, 15]. This could be due to their favorable belief that CAM is less harmful than conventional or mainstream medicine. Many people consider that CAM is as scientific sound as conventional medicine which may persuade them to use it more frequently [6, 13].

### Limitations

There are a few limitations of the study. The study population was drawn from an out-patient department of a medical college hospital in Dhaka, Bangladesh that only offers CAM treatment. Furthermore, this study was cross-sectional, it is difficult to ascertain causality to any of the factors involved. Further, the use of cardinal-based items may reduce the response validity of the variables studied.

## Conclusions

In comparison to other the South Asian countries, about one-third of patients use CAM exclusively to treat their illnesses in Bangladesh. In our estimation of survey information there was a relationship between the usage of CAM exclusively and age, gender, marital status, and economic status. This study examined how patients in Bangladesh use CAM and how they perceive it. Ayurveda and Unani are the most common CAM practices in Bangladesh. Overall, there was a strong association between CAM utilisation and socioeconomic position. Belief in CAM’s effectiveness, less adverse effects, affordability and lower costs are common reasons for accepting CAM for their illnesses. Furthermore, the majority of the former users were satisfied with CAM therapies and would recommend them to others. A nationwide population-based study is required to be undertaken to understand the exact proportion, patterns, and perception of CAM utilisation among the general population.

## Supplementary Information

**Additional file 1.** Questionnaire

**Additional file 2.** Flow chart of data collection process

**Additional file 3. **Types of CAM treatments

**Additional file 4. **Patients’ satisfaction and willingness to advice CAM to others

## Data Availability

The data are available at Mendeley Data. Shahjalal, Md (2021), “Proportion and associated factors of the utilisation of complementary and alternative medicine exclusively in a hospital in Bangladesh”, Mendeley Data, V2, (http://dx.doi.org/10.17632/jpfj36wyf2.2).

## Abbreviations

CAM: Complementary and Alternative Medicine
OOP: Out-of-Pocket
UHC: Universal Health Coverage
SDG: Sustainable Development Goal
