# Supplementary material for: Proportion and associated factors of the utilisation of complementary and alternative medicine exclusively in a hospital in Bangladesh
Source: BMC Complement Med Ther. 2022 Aug 26;22:225. doi: 10.1186/s12906-022-03709-8 (PMC9414049; doi:10.1186/s12906-022-03709-8)
Supplement: Supplementary file 1 — Additional file 1. Questionnaire [file 12906_2022_3709_MOESM1_ESM.docx]

**Questionnaire**

**Proportion and associated factors of the utilisation of complementary and alternative medicine exclusively in a specialised hospital in Bangladesh**

Dear Participant,

We appreciate your voluntary participation in this survey, which will be beneficial for find out the **Proportion and associated factors of the utilisation of complementary and alternative medicine exclusively in a specialised hospital in Bangladesh**.

All the information given by you will be kept confidential. Your identity will not be disclosed. Only study-related personnel will be allowed to see your information.

Please, read the instructions very carefully, and answer all the statements as accurately as possible.

Investigator:

**Md. Shahjalal**

Department of Public Health,

North South University, Dhaka, Bangladesh

Email: [md.shahjalal3@northsouth.edu](mailto:md.shahjalal3@northsouth.edu)

We, researchers, would like to request you to spend approximately 10 to 15 minutes responding to the questionnaire related to imposter syndrome. If you could kindly assist us with this research, it would be much appreciated. Would you agree to participate in this survey and complete the questionnaire?

Yes No

| **Socio-cultural information** |  | |
| --- | --- | --- |
| ID |  | |
| **S1**. Age (year) |  | |
| **S2.** Sex | 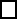 Male 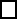 Female | |
| **S3.** Religion | 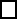 Muslim 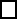 Other | |
| **S4.** Education (highest) | 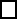 No education 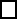 Primary  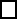 Secondary 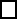 College/University | |
| **S5.** Marital status | 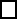 Unmarried 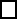 Married (Divorced, Widow) | |
| **S6**. Place of residence | 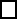 Urban 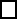 Rural 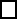 Slum | |
| **S7.** Type of family | 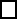 Nuclear 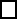 Joint | |
| **S8.** Occupation | 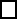 None 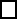 Garments worker  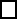 Housemaker 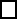 Day Labor  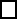 Transport 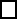 Retired  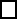 Students 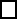 Street Beggar  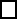 Business 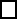 Govt. Job  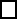 Pvt. Job 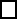 Maidservant  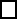 Armed Forces 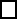 Others…………… | |
| **S9.** Monthly Family income (thousand) |  | |
| **S10.** Smoking history | 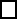 Yes 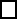 No | |
| **Clinical information** |  | |
| **C1**. Type of disease you have | 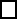 CVS disorders 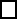 Respiratory disease  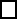 Renal disorders 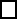 GI disorders  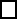 Hepatic disorders 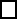 Skin disorders  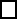 Menstrual disorders 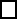 Diabetes  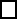 Arthritis 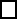 Sexual diseases  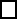 Chronic Pain 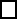 Sinusitis  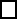 Piles 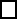 Hypertension  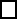 Obesity 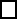 Musculoskeletal disorders  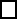 Infertility 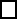 Neurological disorders  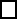 Others………………………… | |
| **C2.** Did you use modern medicine for this disease before? | | 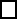 Yes 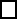 No |
| **C3.** Now, are you using CAM? | | 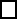 Yes 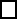 No |
| **C4.** Types of CAM treatment he/she visited: | | 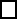 Ayurveda 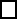 Unani 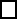 Acupressure  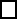 Acupuncture 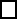 Yoga |
| **C5**. Who/what influences you to use CAM? | | 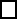 Friends 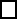 Neighbors  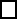 Relative/Family 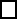 Physician  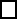 Pharmacist 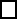 Internet  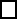 Others |

| **Reasons for using CAM** | [Tick, please] |
| --- | --- |
| **R1.** Lack of trust of modern medicine |  |
| **R2.** Accessibility and availability |  |
| **R3.** Believe CAM is effective |  |
| **R4.** Affordability and lower costs |  |
| **R5.** Believed that CAM had fewer side effects |  |
| **R6.** Avoided of surgery |  |
| **R7.** Advice from family or friends |  |
| **CAM users’ behaviors (Who ever used CAM)** | |
| **B1.** Are you satisfied with the CAM products? | 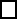 Yes 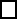 No |
| **B2.** Will you advise others to use CAM for similar problem? | 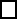 Yes 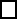 No |

Thank you for your contribution.
