# Supplementary figures and images for: Proportion and associated factors of the utilisation of complementary and alternative medicine exclusively in a hospital in Bangladesh
Source: BMC Complement Med Ther. 2022 Aug 26;22:225. doi: 10.1186/s12906-022-03709-8 (PMC9414049; doi:10.1186/s12906-022-03709-8)

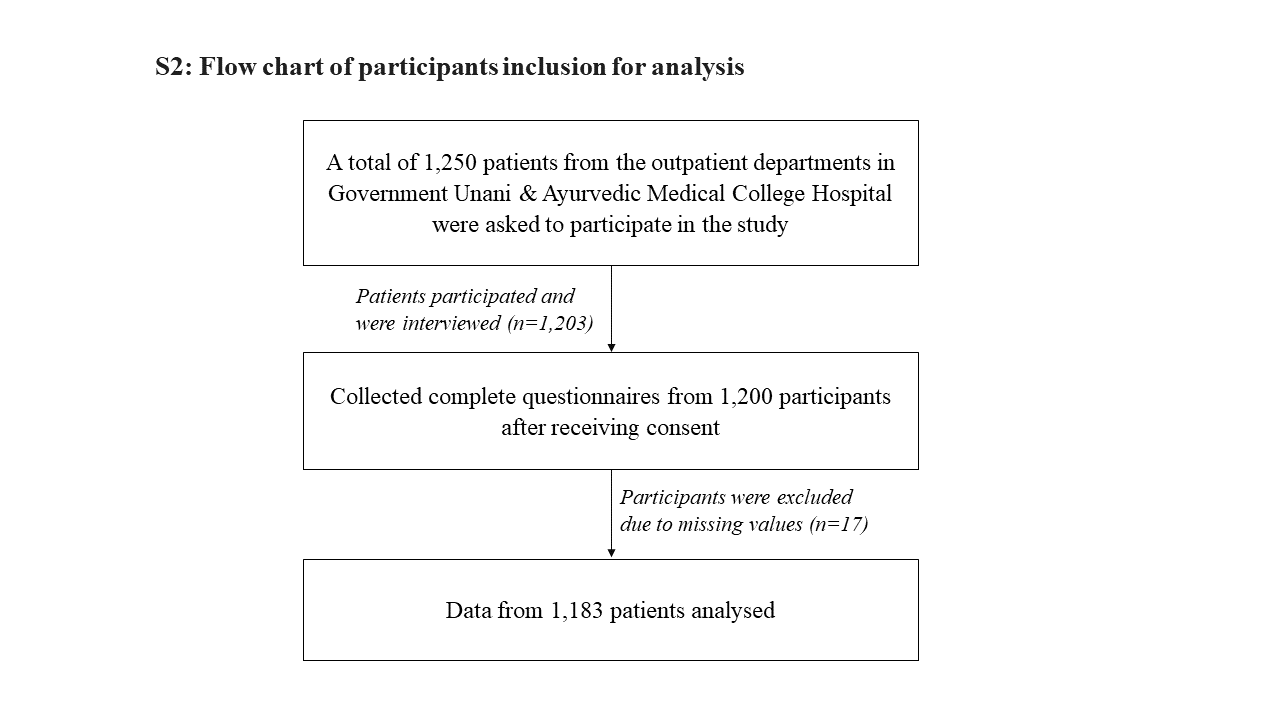

Supplement: Supplementary file 2 — Additional file 2. Flow chart of data collection process [file 12906_2022_3709_MOESM2_ESM.png]

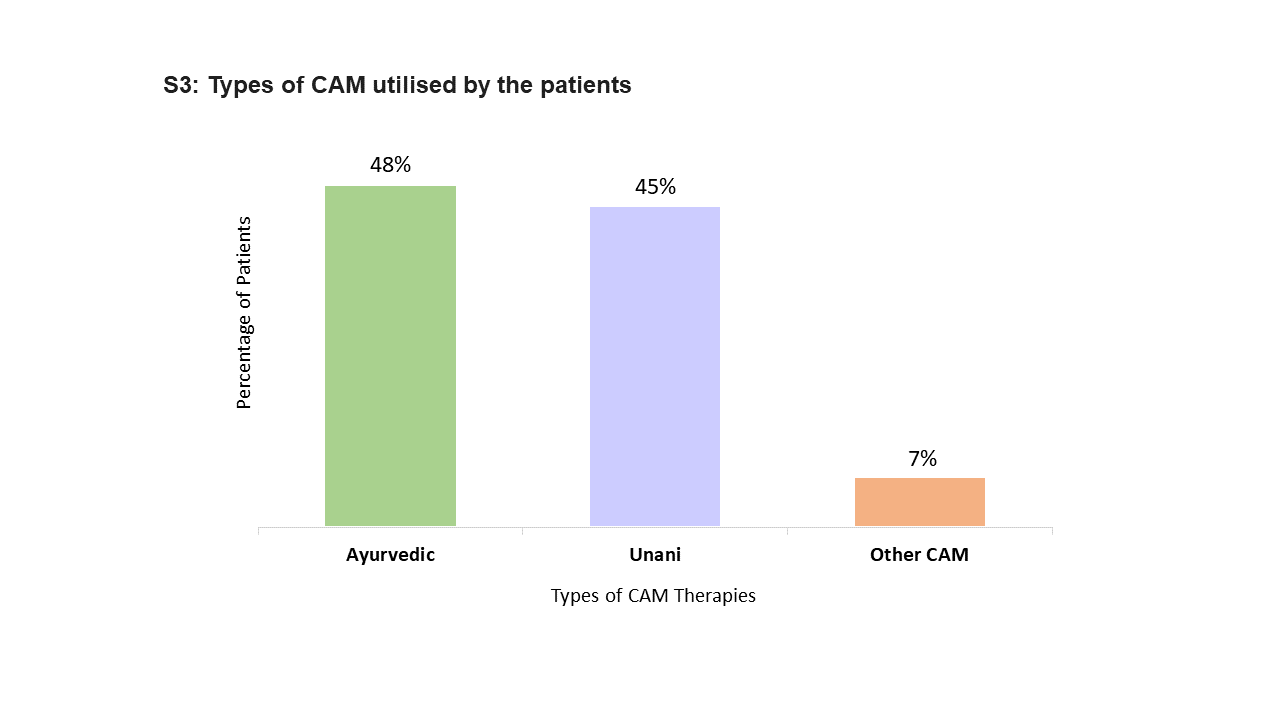

Supplement: Supplementary file 3 — Additional file 3. Types of CAM treatments [file 12906_2022_3709_MOESM3_ESM.png]

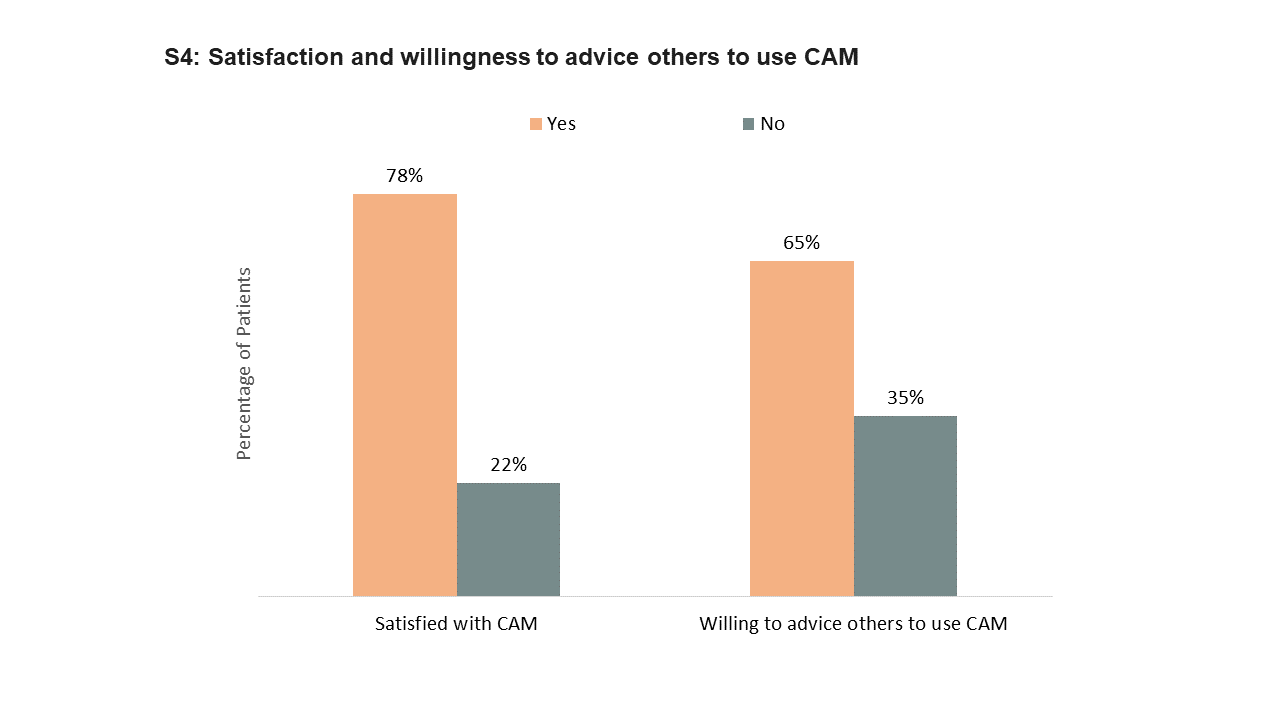

Supplement: Supplementary file 4 — Additional file 4. Patients’ satisfaction and willingness to advice CAM to others [file 12906_2022_3709_MOESM4_ESM.png]
